# Parental satisfaction with paediatric care with and without the support of an eHealth device: a quasi-experimental study in Sweden

**DOI:** 10.1186/s12913-023-10398-7

**Published:** 2024-01-09

**Authors:** Sarah Foldager Jeppesen, Rúnar Vilhjálmsson, Helene Åvik Persson, Inger Kristensson Hallström

**Affiliations:** 1 Department of Health Sciences, Faculty of Medicine, Lund University, Lund, Sweden; 2 Faculty of Nursing, School of Health Sciences, University of Iceland, Reykjavik, Iceland

**Keywords:** eHealth, Health care, Paediatric care, Parents, Quasi-experimental study, Satisfaction, Sociodemographic

## Abstract

**Background:**

The period after a child is discharged from hospital is generally described as challenging for the parents. Their satisfaction with the health services received is an important indicator of the quality of care. eHealth devices are increasingly used in health care to support communication with parents. Differences in levels of parental satisfaction by modality of provided care or by parental background are largely unknown. This study aimed to describe satisfaction with health care between sociodemographic groups of parents, who either received or did not receive an eHealth device for communication between parents and hospital staff as a supplement to routine care after a child’s discharge from neonatal or paediatric surgery departments.

**Methods:**

Data from a quasi-experimental study was collected in the south of Sweden, between 2019 and 2021. The Pediatric Quality of Life Inventory™ (PedsQL) Healthcare Satisfaction Generic Module was used to assess the parents’ satisfaction with different dimensions of health care. Seventy parents of children hospitalized in a neonatal or a paediatric surgery department were enrolled in intervention (eHealth device, *n* = 36) and control (no eHealth device, *n* = 34) groups.

**Results:**

The parents reported high overall satisfaction with the health care provided and were also highly satisfied within different dimensions of care. Moreover, they reported high satisfaction with using an eHealth device, although having support from the eHealth device was related to neither higher nor lower levels of satisfaction with care. There was a significant difference between fathers and mothers in the multivariate sub-analysis in certain instances regarding satisfaction with communication and the level of inclusion.

**Conclusions:**

Parents were very satisfied with the health care provided, whether or not they received eHealth. Further research looking at groups with and without the support of an eHealth device is required to further develop future paediatric and neonatal care interventions. Communication and support through eHealth can be a tool to alleviate the distress parents experience after their child’s hospital admission, accommodate the family’s transfer to home, and increase satisfaction with care, but it needs to be evaluated before being implemented.

**Trial registration:**

Clinical Trials NCT04150120, first registration 4/11/2019.

**Supplementary Information:**

The online version contains supplementary material available at 10.1186/s12913-023-10398-7.

## Background

Specialized paediatric health care for preterm born children and children born with congenital malformations is a continuing challenge for health care systems. The transition from hospital to home can be a challenging period. Having a child at home with a long-term illness or being born preterm is found to be stressful for parents and can impact on their confidence and lead to increased anxiety after discharge [1–3]. In Sweden, highly specialized health care, referred to as National Specialized Medical Care [4], is becoming centralized in a few hospitals in the major cities. A consequence of this centralization is an extended travel distance for families when their child needs specialized care facilities. To increase communication and support at home and to decrease the families’ need for travel to hospitals, different eHealth solutions can be used [5, 6].

eHealth (electronic health) as defined by the World Health Organization (WHO) refers to “the use of information and communication technologies (ICT) for supporting health” and includes a wide range of interventions such as mobile health (mHealth), telehealth and telemedicine [7, 8]. eHealth solutions have been developed and implemented to support equal access to affordable health care and to improve quality of care [8].

The Institute of Medicine (IOM) defines quality of care as the degree to which health services for individuals and populations increase the likelihood of desired health outcomes [9, 10]. The IOM identifies six components of quality in health care services: effective, safe, people-centred, timely, equitable and efficient [10]. In 2018, WHO further added the integrated component into the six components of quality in health care [11]. When studying the quality of provided care, researchers frequently ask patients about their level of satisfaction [12, 13], which is directly linked to the different dimensions of quality of care, and the likelihood that parents will adhere to medical recommendations [14].

Some evidence is found that parental satisfaction with the care and quality of care increases the more the parents collaborate with the health care professionals [15, 16]. A study from South Africa showed that parents were generally very satisfied with the quality of care in a paediatric intensive care unit. The parents were most satisfied with the health care workers’ attitude, but scored information and participation lower [17]. Some of the important determinants of parental satisfaction with care include perceived adequacy of care, health care professionals’ attitude, family support, and parents being an active part in the care of their child [12, 16, 18].

Little is known about sociodemographic differences in parental satisfaction with paediatric hospital care, and the available evidence is mixed. A Greek study of parents of hospitalized children by Tsironi and Koulierakis [16] reported that mothers were less satisfied than fathers with the hospital environment, but other studies of Greek and Norwegian parents of hospitalized children found no significant differences in health care satisfaction between mothers and fathers [19, 20]. Furthermore, a study by Galanis et al. [19] found that older parents were less satisfied with paediatric hospital services than younger parents, but the reverse was found in a study by Hagen et al. [20]. Similarly, a study by Tsironi and Koulierakis [16] found that university-educated parents were more satisfied with parental participation in hospital care than less educated parents, but other studies have found that parents with higher education generally tend to be less satisfied with paediatric hospital care [19, 20]. Finally, there is some limited evidence that parents of foreign nationality are less satisfied with paediatric hospital care than parents with Swedish nationality [19].

High parental satisfaction with using eHealth devices in neonatal and paediatric health care is shown [21]. Recent studies have indicated that parents of children with long-term illness experience access to and use of an eHealth device positively after discharge from the hospital [5, 22]. eHealth in terms of telemedicine is also described as boosting the parents’ sense of self-efficacy, social support, satisfaction, and security [23–25]. It is a preferred communication tool for parents in a home setting [24] and is shown to enhance communication and accessibility between health care providers and parents [25, 26]. Makkar and his colleagues [27] found that parental satisfaction with health care using eHealth communication in video conferencing was high and equivalent to routine care [27], and video communication and web-based eHealth decreased the need for home visits [21]. Thus, it is important to know how to improve the parents’ satisfaction in order to guarantee safe and healthy childcare.

This study aimed to describe parental satisfaction with and without the support of an eHealth device for communication between parents and hospital staff, as a supplement to routine care after a child’s discharge from neonatal or paediatric surgery departments and between sociodemographic groups of parents.

## Methods

### Design

This study is based on quantitative data collection and was part of a larger quasi-experimental study, evaluating a newly developed eHealth device that was provided to parents after discharge from a neonatal or paediatric surgery department in addition to routine care (ClinicalTrials.gov identifier: NCT04150120).

### The eHealth device

The eHealth device is an internet-connected tablet containing a software application developed to enhance and promote parents’ empowerment and self-management and give support to the parents in the transition from hospital to home [28]. Each parent who received the tablet had internet connection through a cryptate line after their child was discharged from the hospital. By using the application, the parents had the opportunity to send and receive text messages, have video conferences, and send pictures to the health care professionals (doctors and nurses) at the hospital through a safe and secure line to support the parents in the care of their child. Furthermore, the parents could register the child’s weight and nutrition status (what the child ate and drank) and send it to the health care professionals.

### Setting and sample

The study took place at the neonatal and paediatric surgery departments in a university hospital in the south of Sweden. Data for the intervention group receiving routine care and support from the eHealth device was collected between November 2019 and October 2021. Data for the control group receiving routine care only was collected between March and July 2020. Inclusion criteria for intervention group: parents of a child under the age of 4 admitted to paediatric care due to premature birth or admitted to paediatric surgery due to surgery in the neonatal period. Inclusion criteria for the control group: parents of a child under the age of 4 who had undergone a surgical procedure. All parents should be able to communicate in Swedish or English. There were 91 parents in the intervention group and 70 parents in the control group who met the inclusion criteria and were asked to participate in the study. Forty-three parents of 33 children consecutively hospitalized due to preterm birth or surgery for congenital malformations that needed surgery were enrolled in the intervention group receiving routine care supported by an eHealth device. Thirty-five parents of 25 children admitted to the paediatric surgery department were consecutively enrolled in a control group receiving routine care after discharge from the hospital. Routine care after being discharged from the neonatal and paediatric surgery department included regular follow-up at the department or out-patient clinics. Furthermore, all the parents were given a name and telephone number they could call if they needed to get in contact with the department between the ordinary appointments.

### Data collection and instruments

Data was collected through online surveys using Research Electronic Data Capture (REDCap).

#### Background and sociodemographic variables

The parents’ background characteristics were collected via a self-report questionnaire which included sociodemographic questions (Supplement 1). Six variables were selected, all of which may affect parents’ satisfaction with the health care received: the parents’ age, gender, marital status, education level, social class, and country of birth. The parents’ age was stratified into four groups, “ ≤ 29”, “30–39”, “40–49”, and “ ≥ 50”, whilst their gender was categorized as “Male” or “Female”. Educational level was assessed as “Compulsory education”, “Vocational education”, “High school”, and “University”. The social class included four distinctions based on the parents’ reported occupational titles: “Lower working-class” (unskilled manual work, e.g. construction workers, house cleaners), “Upper working-class” (craft or trade occupations, e.g. plumbers or carpenters), “Lower middle class” (unskilled or semi-skilled service occupations, e.g. secretaries and office clerks) and “Upper middle class” (professional service occupations, e.g. managers and university-educated specialists) [29]. The variable of where they were born was categorized into two groups, “Outside Sweden” and “In Sweden”. An overview of the parents’ background and sociodemographic data is shown in Table 1.

**Table 1:** Background characteristics and sociodemographic factors of the participants in the intervention and control groups (*N* = 78)

Background variable	Intervention group (*n* = 43)	Control group (*n* = 35)	Total (*N* = 78)
Gender			
Male	18 (41.9%)	15 (42.9%)	33 (42.3%)
Female	25 (58.1%)	20 (57.1%)	45 (57.7%)
Age			
≤ 29	10 (23.3%)	8 (22.9%)	18 (23.1%)
30–39	28 (65.1%)	24 (68.6%)	52 (66.7%)
40–49	4 (9.3%)	3 (8.6%)	7 (9.0%)
≥ 50	1 (2.3%)	0 (0%)	1 (1.3%)
Marital status			
Cohabiting	21 (48.8%)	19 (54.3%)	40 (51.3%)
Married	22 (51.2%)	16 (45.7%)	38 (48.7%)
Education level			
Compulsory education	0 (0%)	1 (2.9%)	1 (1.3%)
Vocational education	2 (4.7%)	3 (8.6%)	5 (6.4%)
High school	13 (30.2%)	9 (25.7%)	22 (28.2%)
University	28 (65.1%)	22 (62.9%)	50 (64.1%)
Social class			
Lower working class	2 (4.7%)	3 (8.6%)	5 (6.4%)
Upper working class	7 (16.3%)	5 (14.3%)	12 (15.4%)
Lower middle class	16 (37.2%)	18 (51.4%)	34 (43.6%)
Upper middle class	18 (41.9%)	9 (25.7%)	27 (34.6%)
Born			
Outside Sweden	3 (8.1%)	4 (11.4%)	7 (9.7%)
In Sweden	34 (91.9%)	31 (88.6%)	65 (90.3%)

#### Assessment of the eHealth device

The parents in the intervention group received three specific questions specially developed for this data collection about their assessment of the eHealth device (Supplement 2) [30]. The following questions were asked using a 5-point Likert scale: What did you think about the access you had to communicate with the health care professionals via the tablet? (range from “bad” to “very good” with “neither good nor bad” in the middle). How satisfied were you with the communication via the tablet from home? (range from “very dissatisfied” to “very satisfied” with “neither satisfied nor dissatisfied” in the middle). How safe did you feel communicating with the health care professionals via the tablet from home? (range from “very unsafe” to “very safe” with “neither safe nor unsafe” in the middle).

#### PedsQL scale dimensions – healthcare satisfaction generic module

The Pediatric Quality of Life Inventory™ (PedsQL) Healthcare Satisfaction Generic Module was used to assess the parents’ level of satisfaction with different dimensions of the health care [31, 32]. This model focuses on satisfaction with health care, both regarding individual dimensions of care and overall. It is based on 24 self-reported items using a 5-point Likert scale with categories ranging from 0 (never) to 4 (always), in addition to a “not relevant” category. A higher score indicates a higher level of health care satisfaction. The 24 questions are composed into six dimensions [31, 33] and linearly transformed to a scale ranging from 0 to 100, where 0 = 0, 1 = 25, 2 = 50, 3 = 75 and 4 = 100 [34].

The PedsQL scale includes six dimensions: 1) Information (five items), 2) Inclusion of family (four items), 3) Communication (five items), 4) Technical skills (three items), 5) Emotional needs (four items) and 6) Overall satisfaction (three items). The Emotional needs dimension was excluded from the study due to less than 50% of items being answered [34].

### Data analysis

A one-way chi-square test was used to assess the frequency distributions of questions relating to the parents’ assessment of the eHealth device. A two-way cross-tabulation and chi-square test was used to assess the relationship between sociodemographic variables and each question concerning parents’ views on the eHealth device. Descriptive statistics presented sample characteristics and the six dimensions of the PedsQL scale, using mean, standard deviation, median, and range. Chronbach’s alpha coefficients were used to measure the internal consistency of each dimension of the PedsQL scale. The Kruskal–Wallis H test was used to test whether the ranked distributions of the dimensional scores were significantly different between the different groups of parents. Eta-squared, adjusted for ordinal data, was calculated to measure effect sizes following the Kruskal–Wallis H test results. Statistical analyses were conducted using RStudio 2021.09.1 + 372 “Ghost Orchid” Release and R 4.1.2, except when calculating Chronbach’s alpha for the six dimensions in the PedsQL Healthcare Satisfaction Module, where the SPSS program was used (IBM SPSS Statistics, version 27.0). Significance was based on a p-value < 0.05. To detect significance for relationships of medium effect size (r = 0.3) with statistical power of 0.8, a total sample size of 85 was required [35]. The total number of participants in the study was somewhat lower (*N* = 78). For this reason, relationships with a p-value between 0.05 and 0.10 were additionally considered as trending toward significance [36].

## Results

In total, 58% of the parents were mothers. Most parents were in the 30–39 age range (67%), most had a university education (64%), and the vast majority had Swedish nationality (93%).

### Assessment of the eHealth device

When asked about the ability to communicate with health care professionals (Table 2), approximately 97% of the parents in the intervention (eHealth) group chose “good” or “very good”, with the vast majority selecting the “very good” category.

**Table 2:** Distribution of the assessment of the eHealth device among the parents from the intervention group (*n* = 36)

**Ability to communicate** **with staff** *n* = 36	**Bad** ***n*** **(%)**	**Less bad** ***n*** **(%)**	**Neither good nor bad** ***n*** ** (%)**	**Good** ***n*** **(%)**	**Very good** ***n*** ** (%)**	**Chi-square value**^**a**^	*p*-value
	0 (0)	0 (0)	1 (2.78)	5 (13.90)	30 (83.30)	41.17	< 0.001
**Satisfaction with communication** *n* = 36	**Very dissatisfied** ***n*** **(%)**	**Dissatisfied** ***n*** **(%)**	**Neither satisfied nor dissatisfied** ***n*** **(%)**	**Satisfied** ***n*** **(%)**	**Very satisfied** ***n*** **(%)**		
	0 (0)	2 (5.56)	4 (11.10)	14 (38.90)	16 (44.44)	16.44	< 0.001
**Security of** **communication** *n* = 36	**Very unsafe** ***n*** **(%)**	**Unsafe** ***n*** **(%)**	**Neither safe nor unsafe** ***n*** **(%)**	**Safe** ***n*** **(%)**	**Very safe** ***n*** **(%)**		
	1 (2.77)	0 (0)	3 (8.33)	13 (36.11)	19 (52.78)	24.00	< 0.001

^a^One-way chi-square test

As for their satisfaction with communication through the eHealth device (Table 2), 83% of the parents reported being either satisfied or very satisfied, although nearly 6% (two parents) stated that they were dissatisfied with the communication.

Regarding security of communication (Table 2), almost 89% of the parents found using the eHealth device “safe” or “very safe”, although about 3% answered “very unsafe”.

One-way chi-square values for the distribution of responses on the eHealth device questions were all statistically significant (Table 2), reflecting the strongly positively leaning responses. A sub-analysis using the chi-square test showed a significant difference by social class in the ability to communicate through the eHealth device, favouring higher social classes (chi-square = 15.94, df = 6, *p* = 0.014). No other significant differences were found in parental views on the eHealth device by sociodemographic variables.

#### Parental satisfaction with and without the support from eHealth device

The mean and median scores on the PedsQL dimensions revealed highly positively skewed data for all parents (Table 3). All the average scores were above 80 (on a scale from 0 to 100), and most of them were close to or around 90. Thus, the scores indicated high overall satisfaction with the different dimensions of health care. Chronbach’s alpha reliability coefficients for the PedsQL dimension were 0.86 for the information dimension, 0.84 for the inclusion dimension, 0.84 for the communication dimension, 0.78 for the technical skills dimension, and 0.88 for the overall satisfaction dimension (Table 3).

**Table 3:** Descriptive statistics among the participants for the five dimensions used from the PedsQL™ healthcare satisfaction module

***PedsQL™ Healthcare Satisfaction Module*** *N* = 70	**Mean (SD)**	**Median [Range]**	**Chronbach’s alpha**
Information (*N*^1^ = 69)	82.19 (18)	85.06 [25–100]	0.86
Inclusion	89.29 (14.45)	93.75 [43.75–100]	0.84
Communication	89.27 (12.88)	93.78 [40–100]	0.84
Technical skills	83.57 (18.44)	91.67 [25–100]	0.78
Overall satisfaction	90.48 (14.42)	100 [33.33–100]	0.88

^1^*N* varies due to missing data in the information dimension

The median values on the PedsQL dimensions for the intervention group were 90 or above for all five dimensions (Table 4). Moreover, the median value for overall satisfaction was 100. Also, all mean scores were above 80 for the intervention group, with the majority scoring around 90. The picture is similar for the control group, except for the information dimension, where average scores tended to be somewhat (albeit not significantly) lower in the control group compared to the intervention group. No significant differences between the intervention and control groups were found in the medians on the five dimensions.

**Table 4:** The differences between five dimensions used from the PedsQL™ healthcare satisfaction module

***PedsQL™ Healthcare Satisfaction Module***	**Control group** *n* = 34		**Intervention group** *n* = 36		*p*-value^**1**^	**Eta-squared**^**a**^
	Mean	Median [Range]	Mean	Median [Range]		
Information	78.18 (n* = 33)	82.34 [25.00, 100] (n* = 33)	85.87	90.00 [50.00, 100]	0.21	0.008
Inclusion	89.45	95.28 [43.75, 100]	89.15	93.75 [50.00, 100]	0.67	0.012
Communication	90.10	92.55 [50, 100]	88.49	95.00 [43.80, 100]	0.75	0.013
Technical skills	83.58	87.56 [33.33, 100]	83.57	91.67 [40.00, 100]	0.56	0.011
Overall satisfaction	91.42	100 [33.33, 100]	89.58	100 [50.00, 100]	0.56	0.011

^a^Eta-squared coefficients are based in the ordinal distribution of scores

^*^ *n* varies due to missing data in the information dimension

^1^Kruskal-Wallis H test

Table 4 also shows small differences in effect sizes in the PedsQL dimensions between parents in the control and intervention groups. The eta-squared effect size coefficients indicated that only approximately 1% (0.8% for information, 1.2% for inclusion, 1.3% for communication, 1.1% for technical skills, and 1.1% for overall satisfaction) of the variation in dimension scores could be associated with membership of the control versus the intervention group.

#### Parental satisfaction between sociodemographic variables

The average median scores for the information dimension were generally high across sociodemographic groups, indicating a high overall level of satisfaction with the information the parents received (Table 5). However, there was a leap in the average values, with a median score of 70.00 for the lower working class and 95.00 or above for the three other social classes, although the class differences were not significant based on the Kruskal–Wallis H test. Also, there were no significant relationships between the information dimension and other sociodemographic background variables.

**Table 5:** Five dimensions from the PedsQL™ healthcare satisfaction module by background and sociodemographic variables

	Information			Inclusion			Communication			Technical skills			Overall satisfaction		
	Median	*p*-value^1^	Eta-squared^a^	Median	*p*-value^1^	Eta-squared^a^	Median	*p*-value^1^	Eta-squared^a^	Median	*p*-value^1^	Eta-squared^a^	Median	*p*-value^1^	Eta-squared^a^
Gender (N* = 70)		0.23	0.007		0.09^+^	0.027		0.09^+^	0.025		0.84	0.014		0.72	0.013
Male (29)	85.00			90.63			92.55			87.50			100		
Female (41)	90.00			93.75			97.55			91.67			100		
Age (N* = 70)		0.15	0.034		0.67	0.022		0.79	0.029		0.46	0.006		0.66	0.021
≤ 29 years (15)	90.00			93.75			95.00			91.67			91.67		
30–39 years (47)	85.00			93.75			92.55			91.67			100		
40–49 years (7)	97.50			92.16			95.05			91.67			95.83		
≥ 50 (1)	100			100			98.73			100			100		
Education level (N* = 70)		0.41	0.002		0.62	0.019		0.43	0.003		0.22	0.022		0.75	0.027
Compulsory education (1)	65.00			93.75			80.00			66.67			83.33		
Vocational education (4)	91.25			95.28			98.78			79.17			100		
High school (19)	90.00			100			95.00			100			100		
University (46)	85.00			93.75			92.55			91.67			100		
Social class (N* = 70)		0.73	0.026		0.68	0.023		0.43	0.004		0.66	0.021		0.38	0.001
Lower working class (5)	70.00			93.75			80.00			75.00			83.33		
Upper working class (10)	97.85			100			93.78			95.89			100		
Lower middle class (30)	95.00			93.75			92.55			91.67			100		
Upper middle class (25)	95.00			93.75			97.55			91.67			100		
Born (N* = 68)		0.35	0.002		0.64	0.011		0.73	0.013		0.66	0.012		0.92	0.015
Outside Sweden (7)	85.00			96.82			97.55			83.33			100		
In Sweden (61)	88.62			93.75			93.78			91.66			100		

^a^Eta-squared coefficients are based on the ordinal distribution of scores

^*^N varies due to missing data

^+^ *p*-values between 0.05–0.10 lean towards significance

^1^Kruskal Wallis H test. *p*-value < 0.05

The average median scores for the inclusion dimension were extraordinarily high (Table 5). All median values were above 90 (on a scale from 0 to 100), showing very high satisfaction with inclusion. However, based on the Kruskal–Wallis H test, there was a trend towards significance of difference in inclusion by gender (*p* = 0.09) with mothers tending to be more satisfied than fathers with the way they were included in the care. Nonetheless, only around 3% of the difference was associated with gender, and both genders still had high scores, showing generally high satisfaction with the inclusion. There were no statistically significant differences between any other sociodemographic groups on this dimension.

There were generally very high median scores for the communication dimension (Table 5). A trend towards significance between fathers and mothers was observed (*p* = 0.09), with mothers tending to be somewhat more satisfied with the communication. Despite this, both genders generally had high scores on satisfaction with communication, with an average score of ≥ 90. None of the other relationships with sociodemographic variables were statistically significant.

For the dimension of technical skills, the picture is largely similar, with generally high median scores, no statistically significant differences, and low eta-squared values (Table 5). Hence, the parents described high satisfaction with the technical skills of the health care professionals.

The median scores for the overall satisfaction dimension also showed very high values (Table 5). Seventy-five percent of the median scores were 100, and the remaining 25% were above 80. Furthermore, the results showed high p-values and low eta-squared values. Thus, overall satisfaction levels were almost close to ideal, with little variation across sociodemographic groups.

Further sub-analyses were conducted to consider whether sociodemographic differences depended on the parents’ receiving eHealth. Regarding inclusion, a significant gender difference within the intervention group appeared (eta-squared = 0.14, *p* = 0.019), indicating that mothers in the intervention group were more satisfied with inclusion than fathers. No significant differences between the remaining groups were observed within the intervention group. Likewise, concerning satisfaction with communication, a significant gender difference within the intervention group was found (eta-squared = 0.12, *p* = 0.023), indicating that mothers were more satisfied with communication than fathers. No other group differences were found. Additionally, a significant difference was found within the control group, with university-educated parents more satisfied with communication compared to parents with lower education (eta-squared = 0.18, *p* = 0.036).

Finally, parental satisfaction with health care professionals’ technical skills differed between educational levels within parents in the control group, with the university-educated respondents being more satisfied (eta-squared = 0.24, *p* = 0.017). No significant differences in satisfaction with technical skills were found between the remaining groups within the intervention and control groups.

## Discussion

Almost all parents of hospitalized children in this study who received an eHealth device for communication with health care staff following their child’s hospitalization thought that their ability to interact with the health care professionals was either “good” or “very good”. Further, the vast majority were either satisfied or very satisfied with the communication through the eHealth device and found that using the device for communicating was “safe” or “very safe”. The parents not receiving support from the eHealth device also reported high levels of satisfaction. There was a significant difference between fathers and mothers, the latter being significantly more satisfied with the inclusion and communication dimensions in the intervention group.

Parents’ assessment of the eHealth device showed high satisfaction levels in all sociodemographic groups. Over 80% of the parents were satisfied with the ability to communicate, with the communication itself, and with its security. This result is in line with other studies on eHealth that generally find a high level of satisfaction with the use of eHealth in health care [37]. Only two parents in this study stated that they were dissatisfied with the communication. Possible barriers and reasons for parents to be less satisfied with communication through the eHealth device could be that parents did not find it flexible and effective to communicate via eHealth, or that it raised some ethical issues [38]. A stable internet connection and essential technical framework are other possible barriers [5, 39].

The security of the eHealth device is critical for patients’ use of eHealth [40]. In the present study 24 parents out of 36 found it “safe” or “very safe” to communicate through the device. The security of using the eHealth device when it comes to the parents, e.g. when sending pictures of the child, is vital and an important ethical consideration in eHealth. Data sharing and security are essential factors for the parents’ satisfaction with care [41].

The results of the current study show generally high levels of parental satisfaction within both intervention and control groups, as well as within different sociodemographic groups. This indicates a high quality of neonatal and paediatric surgery care at the university hospital where the study took place. Few sociodemographic differences in parental satisfaction with care were observed. There were indications that mothers, and parents with higher education and higher reported social class, were somewhat more satisfied on certain dimensions of care, especially within the eHealth (intervention) group. A reason for the mothers being significantly more satisfied with the inclusion and communication dimension could be that women are more knowledgeable about symptoms and treatments compared to men [42, 43], as they are more likely to acquire health-related information through the media and the health care system. Consequently, mothers may be more likely to use and benefit from an eHealth device than fathers. Furthermore, researchers have found that communication between mothers of new-borns and health care professionals is essential for the quality of care [44].

The findings concerning social class and education may suggest that parents with higher education and social class were better able to communicate through the eHealth device, which could imply inequalities in eHealth supported care [45]. Moreover, the digital divide in eHealth could add to social health inequalities [46]. On the other hand, eHealth is often developed to support equity in health and eHealth solutions are an active part of health promotion and attempts to secure good health for all [47]. The results concerning gender and education are partly consistent with one previous parental study [16] but are not compatible at all with other studies [19, 20]. This further indicates that studies of sociodemographic differences in parental satisfaction with paediatric care tend to report conflicting results [16, 19, 20]. This may relate to differences between studies regarding parental characteristics, ages or medical conditions of the children, or the way health services are organized.

Generally, the high levels of satisfaction with care in this study reflect positively on Swedish health care for parents of hospitalized children. Based on earlier research, one would have expected that those who received the eHealth device had a higher level of satisfaction compared to those only receiving routine care. Furthermore, a randomized controlled trial finds that an eHealth application had a statistically significant positive impact on parents’ self-efficacy and satisfaction compared to parents who did not have the application [48].

A Norwegian study within neonatal care found that respect and empathy from the health care workers are key aspects of parental satisfaction [20]. Furthermore, the health care professionals’ attitudes and parents’ involvement in care are some of the key determinants of parental satisfaction [12, 18]. Additionally, the literature finds that a reason why some parents are less satisfied with paediatric care is their unmet need for more training and guidance to take care of their newborn child [20]. Our study suggests that the health care providers accommodated the parents’ needs both with and without the eHealth device.

At children’s hospitals, there is often a clear emphasis on parental involvement [49, 50]. The health care professionals must ensure the child receives proper care and proper supervision. This is where an eHealth device for parent-professional communication can potentially mark a considerable advance in securing good health care. In discussing parents’ role in their child’s care in the face of early discharge, it is essential to recognize the balancing act involved for the health care system. In other words, the system must balance parental willingness to be active parents against limited parental knowledge and skills [51]. Furthermore, parents often have other roles to handle simultaneously: they are parents, workers, have other children to take care of and, of course, they also have obligations to their spouses. It is stated in the literature that taking care of a child that needs attendance and care at home can be demanding and result in parents experiencing a caregiver burden [52]. It is important to balance the parents’ willingness and ability to take care of their family members against their other obligations. The point of the eHealth device used in the current study was to make it easier for the parents to juggle all these balls in the air and reduce hospital visits (e.g., going to the hospital for wound dressing or to measure the child’s weight and height). The trend toward increased parental involvement in paediatric care could affect people unequally, which makes it necessary to consider the capabilities and burdens of different sociodemographic groups.

### Strengths and limitations

The quasi-experimental design allowed this study to compare a treatment and comparison group while including numerous sociodemographic variables, which strengthened the validity of the analysis [53]. However, the comparison group can differ from the intervention group using a quasi-experimental design [54]. To strengthen the internal validity, the strategy was to have rather strict exclusion and inclusion criteria to make the comparison group similar to the intervention group. A strength of the present study is the high percentage (approximately 90%) of enrolled parents that completed all questionnaires. The participants in the dropout group (*n* = 8) were used in a dropout analysis, which showed no statistical differences between the total sample and dropout group regarding sociodemographic variables such as marital status, educational level, and social class.

With the secured eHealth device, the parents could, among other things, exchange text messages and video calls with the health care professionals. Furthermore, an important feature was the parents’ ability to keep track of their children’s development, i.e. the parents could report their children’s weight, height, and nutrition status. The different ways in which parents could use the eHealth device enabled a broad assessment of eHealth in this paediatric context, which can be seen as a strength in the study.

The study used the validated PedsQL Healthcare Satisfaction Generic Module, which has been used previously to describe and measure perceived satisfaction with paediatric health care [55, 56]. The instrument assesses parental overall satisfaction as well as dimensions of satisfaction, and it is a more complete measurement tool than many other measurements of this kind. Cronbach’s alpha coefficients for the PedsQL dimension were all over 0.8 for the information, inclusion, communication, and overall satisfaction dimension and above 0.7 for the technical skills dimension, which is quite satisfactory [57]. Finally, the study used medians due to the presence of outliers and the skewed nature of the data and the Kruskal–Wallis H test as it is quantile-based and therefore not sensitive to outliers.

Our conclusions are limited to communicative eHealth devices of this sort, and do not refer to other eHealth solutions that may also be advantageous, such as general or more specific homepages or apps for parental information on children’s diseases or care. Also, our conclusions refer to parents of children who have undergone hospital treatment, and not to other parents of children receiving health care. Another limitation in the study is the sample size. A limited sample size can threaten the validity and generalizability of a study [53]. Studies sometimes use a level of p between 0.05 and 0.1 in the interpretation of findings to compensate for limited statistical power [58]. This is because only looking at *p* < 0.05 as a significance level runs the risk of a statistical error (beta error), with actual differences in the population being missed. Nevertheless, p-values between 0.05 and 0.1. should be interpreted cautiously. The generalizability of comparing the treatment and control groups can be another limitation of this study, since it did not use random allocation into treatment or control groups [54]. When randomization is not applied, there is a risk of selection bias [53]. The routine care group (control) and the eHealth supported care group (intervention) differed regarding the children’s conditions and treatments. In the intervention group, parents of children from the paediatric surgery and neonatal departments were included, whereas in the control group only parents of children from the paediatric surgery department were included. Finally, the parents were primarily Swedish born with a high educational level, and all of them spoke Swedish or English. Consequently, the results may not be generalizable to other populations of parents of children receiving hospital treatment.

## Conclusions

This study showed high levels of parental satisfaction with paediatric hospital care. The introduction of an eHealth device for communication between parents and health professionals was not associated with lower levels of parents’ satisfaction with care, nor did it substantially increase their satisfaction, despite some specific differences between the mothers and fathers, and by educational levels and social class. eHealth is being used increasingly in the health care sector and could be a tool to alleviate distress among parents. Based on the current study, it is not obvious how and what would need to be done to improve parental satisfaction with care, given the high satisfaction levels, which reflects positively on the Swedish health care system. Parental satisfaction with health care needs to be evaluated with bigger sample sizes and random allocation into eHealth supported care and routine care groups before implementation to further improve paediatric health care.

### Supplementary Information

**Additional file 1. **

**Additional file 2.**

## Data Availability

The data that supports the findings of this study is available from Lund University, but restrictions apply to the availability of this data, which was used under license for the current study, and so is not publicly available. Data is, however, available from I.K-H upon reasonable request and with permission of Lund University.

## Abbreviation

eHealth: Electronic Health
