# Supplementary material for: Parental satisfaction with paediatric care with and without the support of an eHealth device: a quasi-experimental study in Sweden
Source: BMC Health Serv Res. 2024 Jan 9;24:41. doi: 10.1186/s12913-023-10398-7 (PMC10777543; doi:10.1186/s12913-023-10398-7)
Supplement: Supplementary file 1 — Additional file 1. [file 12913_2023_10398_MOESM1_ESM.pdf]

LUNDS UNIVERSITET

Research project: eHealth as an aid for facilitating and supporting self-management in families with long-term childhood illness

<u>Parent</u>

Age:____________________

Gender

- ☐ Male
- ☐ Female

Marital status

- ☐ Cohabiting
- ☐ Married
- ☐ Single parent
- ☐ Divorced/separated
- ☐ Widower/widow

Educational level

- ☐ Compulsory education
- ☐ Vocational education
- ☐ High school
- ☐ University

Occupation:____________________

Born in Sweden

- ☐ Yes
- ☐ No
