# Supplementary material for: Parental satisfaction with paediatric care with and without the support of an eHealth device: a quasi-experimental study in Sweden
Source: BMC Health Serv Res. 2024 Jan 9;24:41. doi: 10.1186/s12913-023-10398-7 (PMC10777543; doi:10.1186/s12913-023-10398-7)
Supplement: Supplementary file 2 — Additional file 2. [file 12913_2023_10398_MOESM2_ESM.pdf]

LUNDS UNIVERSITET

Research project: eHealth as an aid for facilitating and supporting self-management in families with long-term childhood illness

Parents perception of satisfaction and safety regarding communication with staff via e-tablet

Please tick the option that best describes your perception of how communication via the e-tablet worked.

What did you think about the access you had to communicate with the health care professionals via the tablet?

□ Very bad	□ Bad	□ Neither good nor bad	□ Good	□ Very good

How satisfied were you with the communication via the tablet from home?

□ Very dissatisfied	□ Dissatisfied	□ Neither satisfied nor dissatisfied	□ Satisfied	□ Very satisfied

How safe did you feel communicating with the health care professionals via the tablet from home?

□ Very unsafe	□ Unsafe	□ Neither safe nor unsafe	□ Safe	□ Very safe
